# Psychological effects of breastfeeding on children and mothers

**DOI:** 10.1007/s00103-018-2769-0

**Published:** 2018-06-22

**Authors:** Kathleen M. Krol, Tobias Grossmann

**Affiliations:** 1 Department of Psychology, University of Virginia, 485 McCormick Road, 22903 Charlottesville, VA USA; 2 Max Planck Institute for Human Cognitive and Brain Sciences, Leipzig, Germany

**Keywords:** Cognitive development, Brain, Emotion, Oxytocin, Stress, Kognitive Entwicklung, Gehirn, Emotion, Oxytocin, Stress

## Abstract

While the nutritional and physical health benefits of breastfeeding are well established, accumulating research demonstrates the far-reaching psychological effects of breastfeeding on children and their mothers. Here, we provide a non-exhaustive review of the empirical evidence, showing that breastfeeding impacts children’s brain, cognitive, and socio-emotional development. In mothers, research is presented indicating that breastfeeding influences mood, affect, stress, and maternal care. The current review aims to provide a broad overview of existing findings on the psychological effects of breastfeeding, highlighting the important role that breastfeeding plays across several dimensions of psychological functioning. We also discuss the potential mechanisms that may underpin the observed effects, provide a constructive commentary on the limitations of the existing work, and put forth some considerations when evaluating this line of research.

## Zusammenfassung

Während die Vorteile des Stillens im Hinblick auf Ernährung und körperliche Entwicklung gut belegt sind, zeigen Untersuchungen vermehrt die weitreichenden psychologischen Effekte des Stillens auf Kinder und ihre Mütter. Zu diesem Zwecke präsentieren wir einen Überblick der verfügbaren empirischen Befunde, die den Zusammenhang des Stillens mit der hirnphysiologischen, kognitiven und sozialen Entwicklung des Säuglings und Kindes in Verbindung setzen. Außerdem diskutieren wir empirische Untersuchungen zum Einfluss des Stillens auf Mütter mit einem besonderen Fokus auf Emotionen, Stress und mütterliches Verhalten. Dieser Übersichtsartikel vermittelt grundlegende Einblicke in den Stand der Forschung auf diesem Gebiet und unterstreicht die Komplexität des Zusammenspiels von physiologischen und psychologischen Faktoren in der Bestimmung des Einflusses des Stillverhaltens auf Kinder und Mütter. Zusammengenommen deuten die diskutierten Befunde darauf hin, dass das Stillverhalten einen Einfluss sowohl auf die psychologische Entwicklung des Kindes als auch auf das Erleben und Verhalten der Mütter hat. Der aktuelle Review gibt einen Überblick zu existierenden Ergebnissen im Hinblick auf psychologische Einflüsse des Stillens und hebt die wichtige Rolle hervor, die das Stillen in verschiedenen Bereichen psychologischer Funktionen spielt. Zudem diskutieren wir mögliche Mechanismen, die diese beobachteten Effekte stützen können, liefern einen konstruktiven Kommentar zu den Grenzen der vorhandenen Arbeit und bringen Überlegungen zur Evaluation in diesem Forschungsgebiet ein. Für eine exakte Beschreibung und kausale Vorhersage der Effekte des Stillens und damit der Anwendung, bedarf es eindeutig noch weiterer umfangreicher Untersuchungen.

## Introduction

Lactation is a process characteristic of all mammalian species. It is the result of evolutionary forces shaping an optimal nutrient delivery system, involved in supplying all essential nutrients in the adequate amounts from mothers to their offspring [1]. In humans, breastfeeding is undoubtedly the “gold standard” food source in the first months of postnatal life. The World Health Organization and the American Academy of Pediatrics recommend at least six months of exclusive breastfeeding, which is defined by breastmilk as the only source of sustenance [2]. In addition to being a critical source of nutrition to the infant, research shows that breastfeeding is not simply a meal at the breast but also has significant and far-reaching effects on cognition, behavior, and mental health in children and mothers [3]. In this review, we examine existing findings on the psychological effects of breastfeeding in children and mothers. It should be noted that the current review is not exhaustive but is rather designed to provide a broad overview, intended to raise awareness of this growing body of research. Additionally, we discuss potential neurobiological mechanisms that undergird the reviewed psychological effects and point out limitations in the existing research.

Before we begin with the review, it is important to stress that how breastfeeding is measured varies greatly across studies. For example, while some studies treat breastfeeding as a qualitative measure and compare between breastfeeding and bottle feeding, others consider breastfeeding as a quantitative (continuous) variable and measure the duration of exclusive breastfeeding. This fact makes it somewhat difficult to compare studies. We have decided to organize this review according to overarching themes concerning child and maternal psychological effects and to always explicitly mention which breastfeeding measure was used (see *Considerations concerning the effects of breastfeeding on children’s cognitive, social, and brain development* for a discussion on this issue).

## Psychological effects of breastfeeding in children

### Breastfeeding and cognitive outcomes in children

There is a body of research from different countries providing evidence for a link between breastfeeding experience and cognitive development later in life, including improved memory retention, greater language skills, and intelligence [4–9].

Longitudinal prospective designs are a useful method to assess the link between breastfeeding behavior and children’s cognitive development because they do not require retrospective self-report. In one such study, a higher frequency of breastfed meals and the duration of exclusive breastfeeding during the first year of life were found to be positively associated with measures of the Bayley Scales of Infant Development [10], including memory performance, early language, and motor skills at 14 months [11] and 18 months of age [12]. Importantly, these cognitive benefits of breastfeeding seen in infancy have been shown to endure into childhood and adolescence. Specifically, Bernard et al. [13] assessed cognitive and motor development in 2‑ and 3‑year-old children and found that breastfeeding experience was associated with improved cognitive development as measured by the Communicative Development Inventory [14] and Ages and Stages Questionnaire [15]. This study showed that improved problem-solving abilities in children were associated with prolonged duration of exclusive breastfeeding. Similarly, a large population-based cohort study reported significant benefits on executive function (cognitive control) at 4 years of age for those children who were exclusively breastfed for over 6 months after birth compared to those never breastfed as well as those exclusively breastfed for less than 6 months [16]. Quinn et al. [17] followed a cohort from infancy to 5 years of age and found a dose-dependent facilitation of breastfeeding duration on verbal intelligence abilities using the Revised Peabody Picture Vocabulary Test (PPVT-R) [18]. This study showed that at age 5, children who were breastfed for at least 6 months as infants had the highest verbal intelligence scores, while children who were never breastfed had the lowest scores. Another longitudinal study using the Wechsler Intelligence Scale for Children [19] to measure cognitive skills from 1 to 7 years reported persisting cognitive benefits across age as a function of prolonged exclusive breastfeeding duration during infancy [20]. Furthermore, when comparing children who were exclusively breastfed to children who received mixed feeding (formula combined with human milk), the exclusively breastfed children displayed a consistent increase in their intelligence scores from age 1 to age 7. Critically, another large-scale longitudinal study has shown that even when controlling for the intelligence of the mother, intelligence benefits as a function of exclusive breastfeeding experience can be seen among children [21].

The initiation of breastfeeding immediately after birth has also been argued to play a role in reducing the risk for cognitive impairment among children. For example, a clinical study compared the breastfeeding histories of 4‑ to 11-year-old children diagnosed with specific language impairment (SLI) to those of neurotypically developing children and observed that those with SLI were significantly less likely to have been breastfed directly after birth [22]. While this suggests a correlation between early breastfeeding experience and the development of a specific cognitive impairment, it would be premature and problematic to assign any causal influence to the lack of early breastfeeding on a specific cognitive impairment.

More compelling evidence relating breastfeeding to cognitive outcomes comes from a randomized controlled intervention study including over 13,000 mother–infant dyads [7]. In this study, mothers were randomly assigned to an exclusive breastfeeding promotion intervention, which led to a seven-fold increase in exclusive breastfeeding at 3 months of age. In this study, children were longitudinally followed and those children who had prolonged exclusive breastfeeding experience as infants showed higher intelligence scores and higher teacher ratings of academic proficiency at the age of 6.5 years [7]. A recent follow-up study with the same cohort of children at 16 years of age revealed a persistent impact of prolonged exclusive breastfeeding experience on verbal abilities, but not on any other neurocognitive measures [23]. The authors of this study suggest that over time, the effects of breastfeeding may be “diluted”, and other environmental factors such as peer influence and parental intellectual stimulation may become better predictors of cognitive function.

There is, however, some evidence to demonstrate that breastfeeding experience during infancy impacts cognitive abilities well beyond infancy, even into adulthood. For example, Mortensen et al. [4] investigated cognitive performance in two different cohorts using different intelligence tests. This study showed that across cohorts and measurement instruments, longer duration of breastfeeding during infancy was positively associated with cognitive performance as adults [4]. Similarly, recent findings from another cohort revealed that the duration of exclusive breastfeeding was positively associated with increased intelligence, educational attainment, and income at 30 years of age [24]. In fact, there is also work to show that breastfeeding duration during infancy is positively associated with reading ability at 53 years of age, as measured by the National Adult Reading Test [25].

It is crucial to highlight that the aforementioned studies controlled for a large range of potentially confounding maternal variables, including but not limited to education, employment, income, age, method of delivery, cigarette consumption during pregnancy, and infant birth weight. Indeed, one large-scale study, which included a multitude of potential confounds in their analysis such as maternal intelligence quotient (IQ), social class, and education level, as well as less commonly included confounding variables such as maternal psychopathology, attachment, and exposure to pollutants, still found a robust and independent positive impact of prolonged exclusive breastfeeding duration on neuropsychological function in children [16]. Yet, it is important to acknowledge that not all studies find such clear associations between breastfeeding and cognitive outcome measures when controlling for potential confounds. For example, a study by Jacobson et al. found an initial impact of breastfeeding on children’s intelligence scores at both 4 and 11 years of age, but this effect was much reduced when adjusting for maternal intelligence and parenting skills assessed during home observations using the Home Observation Measurement of the Environment (HOME) [26]. Similarly, when controlling for socio-economic status and gestational age, von Stumm and Plomin [27] report only a marginal impact of breastfeeding experience on girls’, but not boys’, IQ at 2 years of age and no impact at a follow-up visit at 16 years. More generally, due to the high number of potentially confounding factors and the difficulty of controlling for all of them effectively in one study, caution is needed when designing and interpreting studies investigating the effects of breastfeeding on cognitive development [28, 29]. For a systematic and informative review of the role of confounding variables in breastfeeding research, see [30]. Nonetheless, the existing evidence reviewed in this section points to a beneficial effect of breastfeeding, especially prolonged exclusive breastfeeding, on children’s cognitive (intellectual) development.

This raises the question of what mechanism underpins these effects of breastfeeding on cognitive development. One possible mechanism may relate to specific nutrients such as the long-chain polyunsaturated fatty acids (LC-PUFAs), which are present in human milk but usually absent in formula [31]. Two major LC-PUFAs are docosahexaenoic acid (DHA) and arachidonic acid (ARA), which are involved in neurodevelopment by contributing to healthy neuronal growth, repair, and myelination [32]. Importantly, myelination predominately occurs postnatally within the first 18 months of life [33, 34]. Infants produce a small quantity of DHA during the first 2 weeks of life, but are then unable to produce sufficient amounts on their own until about 6 months of age [34]. This suggests the possibility of a window in development during which human brain and cognitive development may be particularly sensitive to LC-PUFAs supplied through breastfeeding.

There is evidence to support the importance of LC-PUFAs as contributors to cognitive development. For example, Caspi et al. [35] investigated how individual differences in the ability to metabolize and produce LC-PUFAs influences the impact of breastfeeding on cognitive development. More specifically, they assessed two single-nucleotide polymorphisms (SNPs) on the *FADS2* gene (rs174575 and rs1535), which encodes an enzyme that directly impacts metabolism of DHA and ARA. Children who were breastfed displayed higher intelligence scores from ages 5–13 years, in line with the aforementioned studies. Critically, *FADS2* genotype further impacted this association such that breastfed carriers of the C allele on rs174575, associated with more efficient processing of fatty acids (i. e., LC-PUFAs), had the highest intelligence scores overall. This suggests that the impact of breastfeeding on cognitive development is greater among individuals genetically predisposed to more efficiently process LC-PUFAs. Additionally, there is evidence that formula supplemented with DHA can improve cognitive development [31]. Taken together, research reviewed in this section attests to the impact of breastfeeding on cognitive development and highlights potential mechanisms accounting for such effects. The next section will review existing research on how breastfeeding experience influences brain development during infancy and thereby helps us to better understand how breastfeeding impacts cognitive development.

### Breastfeeding and brain development in children

Research into the potential impact of breastfeeding on brain development complements and extends work on cognitive development by using methodologies such as electroencephalography (EEG) and magnetic resonance imaging (MRI). One such study measured EEG spectral power longitudinally over the course of the first year of life in a group of typically developing infants and compared between breastfed and formula-fed infants [36]. This study showed that, within the frequency range thought to be most impacted by myelination (0.1–3 Hz), formula-fed infants displayed an earlier peak (at 6 months) than breastfed infants (at 9 months) in EEG power measured in this frequency range followed by a decline with age seen in both groups. This study suggests that breastfeeding influences the timing of myelination processes in the developing infant brain by prolonging the peak of myelination to a later age. While the authors of this study make no strong claims regarding a benefit of breastfeeding, they suggest that these different patterns of early neurodevelopment may set off differential trajectories in brain and cognitive development between breastfed and formula-fed infants.

Studies employing structural and diffusion-weighted MRI critically complement and extend the above-mentioned findings by directly measuring differences in brain structure. In line with the finding that breastfeeding impacts the timing of myelination, whole brain volume, cortical thickness, and white matter volume have all been found to be increased among children with longer durations of breastfeeding experience [33, 37–39]. For example, in a cross-sectional design, Deoni et al. [33] investigated white matter maturation from 10 months to 4 years of age and found a positive association between the duration of exclusive breastfeeding and the development of white matter tracts. This study reported breastfeeding-related increases in white matter in regions that typically mature later in development, including frontal and temporal regions. Furthermore, this study reported that breastfeeding was associated with white matter in tracts commonly associated with higher-order cognition and socio-emotional functioning, including the superior longitudinal fasciculus [33]. Another critical follow-up study from the same group of researchers assessed changes in white matter volume in a longitudinal design [39]. In this study, breastfed children displayed a prolonged window of white matter development between 16 months and 2 years, resulting in an overall myelin increase detectable by 2 years of age that persisted through childhood. These findings corroborate the EEG spectral power analyses presented above [36], suggesting that breastfeeding influences the timing and duration of myelination processes in infancy. In comparison, formula-fed infants displayed a significantly slower rate of white matter development between 1 and 2 years of age, and the overall volume continued to remain below the volume measured for the breastfed infants. Furthermore, Deoni et al. [39] compared the brain development outcomes of infants fed different types of formula. Notably, infants fed with formulas with the highest levels of DHA and ARA showed the white matter development most similar to breastfed infants, albeit on a smaller scale. This suggests that adding DHA and ARA to formula can help reduce the effect that the absence of breastfeeding has on white matter development during infancy. At the same time, this study also shows that adding DHA and ARA to formula cannot completely restore the effects of breastfeeding, suggesting that there are other factors at play that contribute to the effects of breastfeeding on brain development.

Taken together, these findings regarding brain development suggest that elements of breast milk itself, particularly LC-PUFAs, likely contribute to enhanced patterns of myelination in the developing brain, but they do not fully account for the reported effects of breastfeeding on brain development. Therefore, there must be additional factors that contribute to the seen effects of breastfeeding. Such factors could potentially be aspects of the interaction between mother and infant such as touch and warmth, or other substrates contained in the breastmilk such as hormones that are not present in formula.

### Breastfeeding and social and emotional development in children

In addition to the effects reported on children’s cognitive and brain development, there is evidence that breastfeeding also impacts social and emotional development in children. There is work to suggest that breastfeeding experience is associated with differences in infant temperament. For example, at 3 months of age, breastfed infants are reported to show greater negative affect than formula-fed infants [40]. Similarly, negative temperament, such as fussiness, has also been found to be associated with a prolonged duration of breastfeeding in infancy [41]. In contrast, another study found that breastfed infants were reported to have more “vigor” at 3 months of age, characterized by greater approach and activity, than formula-fed infants [42]. Thus, the evidence concerning the association between breastfeeding is mixed and may depend on the specific temperament characteristic examined. There is also research indicating a negative association between breastfeeding experience and aggressive behavior. For example, duration of breastfeeding experience has been shown to correlate negatively with parent-reported antisocial and aggressive behavior in children from 4 to 11 years of age [43]. These effects on antisocial behavior appear to extend well beyond childhood into adulthood. A longitudinal study following adults from 20 to 40 years of age found significantly greater amounts of hostile (aggressive) behavior in adults who were not breastfed as infants compared to those who were breastfed [44].

Furthermore, there is accumulating evidence to suggest that the absence or short duration of exclusive breastfeeding might be associated with the development of autism spectrum disorder (ASD), a neurodevelopmental disorder characterized by social impairments. A recent meta-analysis of over 2000 children reports that those diagnosed with ASD were significantly less likely to have been breastfed than neurotypical children [45]. Furthermore, it has been reported that children with over 6 months of exclusive breastfeeding or formula supplemented with DHA exhibit the lowest probability (measured as odds ratios) for subsequently being diagnosed with ASD [46]. Along the same lines, Al-Farsi and colleagues observed that exclusive breastfeeding duration significantly reduced the likelihood for developing ASD. This study further reported that the late initiation of breastfeeding increases likelihood for developing ASD, possibly related to the limited or lacking consumption of colostrum or first milk by the newborn infant, which is particularly rich in antibodies, immune cells, and protein content [47].

It is important to emphasize that some studies have not found an impact of breastfeeding on ASD diagnosis. For example, in a large phone survey of parents of 2‑ to 5‑year-old children, ASD diagnosis was not associated with any measure of breastfeeding history, including exclusive breastfeeding duration [48]. It is also critical to note that it is problematic to assign a causal role to breastfeeding in the development of ASD because infants later diagnosed with ASD as children may already display certain characteristics that make breastfeeding more difficult for the mothers. A study by Lucas and Cutler reports “dysregulated” breastfeeding patterns in infants later diagnosed with ASD, and cite potential mechanisms for atypical feeding patterns such as reduced joint attention during social interactions [49]. More generally, large prospective longitudinal studies that measure social development directly (experimentally) and comprehensively in children are needed to appropriately address this issue.

Empirical investigations into how breastfeeding experience impacts responses to social information processing during infancy have only recently been introduced. For example, Krol et al. [50] examined how exclusive breastfeeding duration affects infants’ brain responses to emotional body cues using event-related potentials (ERPs). This study showed that 8‑month-old infants who had been breastfed for longer durations (more than 5 months) displayed an enhanced attentional brain response to happy expressions while reducing attention to fearful expressions, suggesting that longer exclusive breastfeeding experience is associated with a greater attentional bias to positive emotion. Similarly, in another study using eyetracking with 7‑month-old infants, exclusive breastfeeding duration was associated with an increased attention to happy eyes and reduced attention to angry eyes [51]. Furthermore, the effect of breastfeeding depended upon genetic variation within the endogenous oxytocin system as indexed by a common SNP (rs3796863) on the gene encoding CD38, an ectoenzyme that mediates the release of oxytocin. This study showed that infants with the genotype linked to decreased levels of oxytocin and increased risk for ASD (CC genotype) [52, 53] were most strongly impacted by the duration of exclusive breastfeeding experience. These findings from experimental work with typically developing infants show that individual variability in responding to emotional information is systematically linked to breastfeeding and might depend on endogenous factors related to the oxytocin system. It is thus possible that endogenous (genetic) and exogenous (breastfeeding) factors influencing the developing oxytocin system are at least partly responsible for shaping socio-emotional development in children.

### Considerations concerning the effects of breastfeeding on children’s cognitive, social, and brain development

In general, breastfeeding experience has been associated with improved cognitive abilities, facilitated brain development, and a reduced risk for antisocial behaviors and atypical social development including ASD. However, there are several issues to keep in mind when considering this line of research.

First, breastfeeding as the independent variable is often measured differently across studies, which makes it difficult to compare between studies. Specifically, many of the studies reviewed above analyzed breastfeeding experience as a dichotomous categorical measure (qualitative)—breastfeeding versus no breastfeeding, whereas other studies employed a continuous (quantitative) breastfeeding measure such as the duration of exclusive breastfeeding, or the current percentage of meals still breastfed. Yet another set of studies used the timing of breastfeeding initiation and found that this critically contributes to the effects on certain outcome measures [54]. Given this issue, research is needed that compares these different measures of breastfeeding experience in order to better understand the exact relation between breastfeeding, its duration, and timing with the critical outcome measures regarding children’s development. Second, there is an issue concerning the specificity of the effects of breastfeeding that can be concluded from the reviewed studies. To date, there is no research that examines the effects of breastfeeding including brain, cognitive, and social development measures of children within the same study. In other words, research that examines multiple dependent variables combining brain, cognitive, and social data about children’s development is needed. Third, we are only beginning to elucidate the physiological (neurobiological) mechanisms that underpin the psychological (cognitive and social) effects seen in children.

With respect to those underlying mechanisms, we would like to briefly outline a working model as to how breastfeeding impacts child development (see Table 1). Based on the research reviewed above, we suggest the following two key processes to account for (a) cognitive development benefits and (b) social development benefits as they are related to breastfeeding. A: The LC-PUFAs contained in human breast milk critically contribute to white matter development during childhood which accounts for improved cognitive and intellectual functioning. B: Oxytocin contained in human breastmilk and further released during breastfeeding through suckling, touch, and warmth facilitates socio-emotional functioning in the infant by enhancing positive tendencies (approach) and reducing negative tendencies (withdrawal and anxiety). This likely accounts for improved social development and reduced antisocial and atypical social behaviors.

**Table 1:** Working model^a^ of how breastfeeding may impact neurocognitive and socio-emotional outcomes in children and mothers

	Breastfeeding substrate	Source	Purported Mechanism	Outcome
*Child*				
Neuro-cognitive	LC-PUFAs (i. e., DHA and ARA)	Breast milk Genetic variation	Neuronal growth and repair Myelination	Extended rate and duration of myelination Increased whole brain volume and cortical thickness Increased white matter volume Heightened cognitive performance (i. e., IQ, executive function)
Socio-emotional	Oxytocin	Breast milk Endogenous release due to touch, warmth, and eye contact during social interaction Genetic variation	Facilitated social perception Prosocial behavior Bonding Anxiolytic effects Interaction with other hormones and neurotransmitter systems	Heightened attention to positive emotional expressions Reduced antisocial and aggressive behavior Reduced likelihood of ASD diagnosis
*Mother*				
Socio-emotional	Oxytocin	Milk ejection reflex Endogenous release due to touch, warmth, and eye contact during social interaction Genetic variation	Facilitated social perception Prosocial behavior Bonding Anxiolytic effects Interaction with other hormones and neurotransmitter systems	Reduced subjective stress Reduced physiological stress (i. e., cortisol levels, cardiac vagal tone modulation) Mother–infant attachment Heightened neural sensitivity to infant cues Reduced postpartum depression Heightened positive affect

*LC-PUFAs* long-chain polyunsaturated fatty acids, *DHA* docosahexaenoic acid, *ARA* arachidonic acid, *IQ* intelligence quotient, *ASD* autism spectrum disorder

^a^Here we outline potential mechanisms underlying the main psychological effects observed in this review. Please note that this list is not exhaustive and only serves to highlight potential underlying processes and mechanisms

## Psychological effects of breastfeeding in mothers

### The impact of breastfeeding on affect, mood, and stress in mothers

Breastfeeding has been reported to impact mood and stress reactivity in mothers [55]. Specifically, breastfeeding mothers report reductions in anxiety, negative mood, and stress when compared to formula-feeding mothers [56]. These findings based on subjective self-report measures are supported by objective physiological measures indicative of a positive effect of breastfeeding on emotional well-being. For example, breastfeeding mothers have stronger cardiac vagal tone modulation, reduced blood pressure, and reduced heart rate reactivity than formula-feeding mothers have, indexing a calm and non-anxious physiological state [57, 58]. Moreover, there is evidence to show that breastfeeding mothers have a reduced cortisol response when faced with social stress [55]. Breastfeeding mothers also display prolonged and higher quality sleep patterns than those who feed their infants formula. Specifically, there is research to show that at 3 months postpartum, breastfeeding was associated with an increase of about 45 minutes in sleep and reduced sleep disturbance [59]. Critically, breastfeeding also impacts mothers’ responses to emotions in others and may thereby improve social interactions and relationships. More specifically, recent work shows that prolonged durations of exclusive breastfeeding are linked to facilitated responses to inviting (happy) facial expressions and that more frequent breastfeeding on a given day is linked with reduced responsivity to threatening (angry) facial expressions [60].

In summary, there is research showing that breastfeeding has beneficial effects on mothers’ own mood, affect, and stress, and also that breastfeeding facilitates responses to positive emotions in others. Similar effects on affect and stress as seen here for breastfeeding are also observed in studies administering oxytocin intranasally compared to a placebo [61, 62], suggesting that breastfeeding may affect (increase) endogenous oxytocin levels in the mothers. This is in line with the known role of oxytocin during breastfeeding and supported by research documenting a rise in maternal oxytocin levels during breastfeeding [63]. More evidence in support of this notion comes from a recent study which revealed that mothers’ genetic variation in oxytocin (as indexed through the *CD38* rs3796863 SNP) impacts the rate at which cortisol decreases during a breastfeeding session. Specifically, mothers with the non-risk genotype, associated with higher oxytocin levels, showed a steeper reduction in cortisol. Strikingly, this differential reduction in cortisol was found in their infants as well [64]. It is thus likely that the positive effects of breastfeeding on the measures reviewed above have a physiological basis in an upregulation of endogenous oxytocin levels among breastfeeding mothers.

### Breastfeeding and mother–infant attachment

Breastfeeding is also thought to facilitate maternal sensitivity and secure attachment between mother and child [65–67]. There is research to show that mothers who breastfeed tend to touch their infants more [68], are more responsive to their infants [69], and spend more time in mutual gaze with infants during feedings than bottle-feeding mother–infant dyads do [70]. Moreover, in a prospective longitudinal study of 675 mother–infant dyads, increased duration of breastfeeding was associated with maternal sensitive responsiveness, increased attachment security, and decreased attachment disorganization when infants were 14 months of age [71]. Brain imaging work also provides evidence for a positive influence of breastfeeding on the mother–child relationship. For example, in a functional MRI (fMRI) study, it was found that exclusively breastfeeding mothers exhibited greater brain activation in several limbic brain regions when listening to their own infant’s cries as compared to exclusive formula feeders, suggesting greater involvement of emotional brain systems in breastfeeding mothers [72].

In this context, it is important to note that breastfeeding has not always been found to be directly linked to attachment quality [73]. For example, Britton et al. [74] did not find an association between breastfeeding experience and mother-infant attachment at 12 months. However, this study did find that maternal sensitivity at 3 months of age significantly predicted the duration of breastfeeding during the first year of life. Additionally, maternal sensitivity in other studies has been linked to improved attachment quality [75]. Taken together, these findings suggest that the association between breastfeeding and attachment quality might be at least partly accounted for by more direct effects of breastfeeding on maternal sensitivity. This possibility is also supported by the findings reported above, indicating that breastfeeding mothers display more positive mood, less stress, and more effective emotional responding to others, which is likely to positively influence their maternal behaviors [55, 60].

### Breastfeeding and postpartum depression

There is a growing body of evidence indicating that breastfeeding behavior is linked to postpartum depression in mothers [76, 77]. Hamdan and Tamim [78] showed in a prospective study that breastfeeding mothers had lower scores on the Edinburgh Postnatal Depression Scale (EPDS) at 2 and 4 months postpartum and were less likely to be diagnosed with postpartum depression at 4 months postpartum. Moreover, this study revealed that higher depression scores at 2 months postpartum were predictive of lower rates of breastfeeding at 4 months. In another prospective study, a significant decrease in depression scores was observed from the third trimester of pregnancy to 3 months postpartum in mothers who exclusively breastfed for more than 3 months when compared to mothers who breastfed for less than 3 months [2]. Importantly, this study showed that depression scores during the third trimester of pregnancy were linked to decreased exclusive breastfeeding duration postpartum, suggesting that maternal mood and affect predicts breastfeeding behavior in mothers.

Considering the complicated and potentially reciprocal association between breastfeeding and maternal depression, it is also possible that issues with breastfeeding, which may lead to earlier cessation of breastfeeding, could impact maternal mood and affect. For example, Brown et al. [79] found that breastfeeding cessation is correlated with high depression scores in mothers, but when examining this correlation more closely found that it was only present in mothers who stopped breastfeeding due to physical difficulty and pain when breastfeeding. Another study assessed breastfeeding complications and maternal mood at 8 weeks postpartum and found that breastfeeding problems alone, or co-morbid with physical problems, were associated with poorer maternal mood [80]. These findings highlight the importance of understanding the exact nature of problems with breastfeeding and also mothers’ reasons for ceasing to breastfeed, and how this impacts mood and affect in mothers, when studying the link between breastfeeding and postpartum depression. While breastfeeding is associated with maternal mood and postpartum depression, it is difficult to know whether it is breastfeeding or maternal mood or affect that is driving (causing) the effects due to the complex relation between breastfeeding and maternal mood and affect. For example, there is evidence to suggest that mothers with higher levels of anxiety and depression display reduced exclusivity and quicker cessation of breastfeeding, as well as a more negative attitude towards breastfeeding [81, 82]. Nonetheless, the observed association between breastfeeding and depression is broadly in line with what is mentioned above regarding the effects of breastfeeding on maternal affect, mood, and stress.

## Conclusions

The current review provides an overview of the critical and far-reaching psychological effects of breastfeeding in children and their mothers, and proposes potential physiological bases (substrates) accounting for these effects. In children, breastfeeding has been associated with improved cognitive performance and socio-affective responding. Improved cognitive performance in children is likely linked to the fatty acids (i. e., LC-PUFAs) contained in breastmilk and their potential beneficial effect on brain development during infancy, especially concerning the growth of white matter tracts (myelination). Heightened socio-affective responding seen in breastfed children is possibly connected to the stimulation of the oxytocin system and oxytocin’s known role in promoting positive affect and approach behaviors, while reducing stress and avoidance behavior. In mothers, breastfeeding significantly reduces physiological and subjective stress, facilitates positive affect, and improves maternal sensitivity and care. Again, the oxytocin system likely plays an important role in explaining the effects on maternal psychology and behavior.

In this context, it is important to acknowledge that the proposed framework of how to conceptualize the effects of breastfeeding on mothers and children does not fully capture the highly complex and interactive nature of how breastfeeding affects both the mother and the child. In fact, research is urgently needed to empirically address this issue by simultaneously studying the psychological effects in both mothers and their children in large-scale, prospective longitudinal designs with physiological measures. To undertake such comprehensive research in the future seems imperative given not only its potential for improving mental health of children and their mothers, but also because of its implications for clinical practice and social policy.
